# Cognitive Function and Whole-Brain MRI Metrics Are Not Associated with Mobility in Older Adults with Multiple Sclerosis

**DOI:** 10.3390/ijerph18084232

**Published:** 2021-04-16

**Authors:** Jessica F. Baird, Robert W. Motl

**Affiliations:** Department of Physical Therapy, University of Alabama at Birmingham, Birmingham, AL 35294, USA

**Keywords:** mobility, aging, multiple sclerosis

## Abstract

Due to advances in disease-modifying medications and earlier management of comorbidities, adults with multiple sclerosis (MS) are living longer, and this coincides with the aging of the general population. One major problem among older adults with and without MS is limited mobility, a consequence of aging that often negatively affects quality of life. Identifying factors that contribute to mobility disability is needed to develop targeted rehabilitation approaches. This study examined cognitive processing speed and global brain atrophy as factors that may contribute to mobility disability in older adults with and without MS. Older adults (≥55 years) with MS (*n* = 31) and age- and sex-matched controls (*n* = 22) completed measures of mobility (Short Physical Performance Battery) and cognitive processing speed (Symbol Digit Modalities Test) and underwent an MRI to obtain whole-brain metrics (gray matter volume, white matter volume, ventricular volume) as markers of atrophy. Mobility was significantly worse in the MS group than in the control group (*p* = 0.004). Spearman correlations indicated that neither cognitive processing speed (MS: r_s_ = 0.26; Control: r_s_ = 0.08) nor markers of global brain atrophy (MS: r_s_ range = −0.30 to −0.06; Control: r_s_ range = −0.40 to 0.16) were significantly associated with mobility in either group. Other factors such as subcortical gray matter structures, functional connectivity, exercise/physical activity, and cardiovascular fitness should be examined as factors that may influence mobility in aging adults with and without MS.

## 1. Introduction

There are an estimated 1 million adults living with multiple sclerosis (MS) in the United States (US), and 43% of them are 55 years of age or older [1]. This reflects an age-related shift in the prevalence of people with MS associated with the “greying” of the general US population and major advances in disease-modifying medications that extend life expectancy in MS. Indeed, the life expectancy of adults with MS (75.9 years) is reportedly 7.5 years less than that of adults without a chronic neurological disease (83.4 years) [2].

One major problem among older adults with and without MS is declining mobility [3,4,5]. This is one of the most debilitating consequences of aging that negatively impacts quality of life [6,7]. However, unlike the general population, older adults with MS face the combined burden of aging and living with a chronic neurological disease. Indeed, one study of 353 adults with MS over the age of 55 years reported that 86.4% of participants used at least one device to assist with mobility [8]. Additionally, strategies for improving mobility among adults with MS, such as medications (i.e., dalfampridine), exercise training, and gait training, have yielded inconsistent results [9]. This underscores the need for effective rehabilitation approaches for managing mobility disability in adults with MS. The intersection of aging and MS may have unique factors underlying mobility disability compared with aging alone. An important first step for developing targeted rehabilitation approaches that prevent and/or remediate the detrimental effects of aging, with or without MS, on mobility requires examining correlates of mobility disability.

There is evidence in adults with MS that the decline in cognitive function may be associated with impaired mobility. Several studies in adults with MS have demonstrated an association between worse cognitive functioning (often indicated by impaired cognitive processing speed) and worse walking performance (i.e., walking speed, walking endurance, self-reported walking ability) [10,11]. Additionally, cognitive processing speed is a significant predictor of lower motor function in adults with MS [12]. This is further supported through the application of dual-task walking paradigms that demonstrate that walking performance is diminished when simultaneously performing a cognitive task, and this suggests that cognitive difficulties may interfere with mobility [13]. Together, evidence from dual-task paradigms and from comparatively younger adults with MS suggests that cognitive function, and more specifically cognitive processing speed, may contribute to impaired mobility.

The potential effect of cognitive functioning on mobility in aging individuals further suggests that other central factors such as brain structure might affect mobility as people age. The overlap of cognitive and physical decline that is associated with aging indicates there may be a common source for this concurrent deterioration [14]. Conceptual models describing cognitive-motor interference suggest that, as both cognitive function and motor function are centrally mediated processes, brain structure may significantly affect mobility as people (with or without MS) age [15,16]. Among adults with MS where brain atrophy occurs at a higher rate than the healthy aging population [17], different measures of brain atrophy, such as decreased white and gray matter volume and ventricular enlargement, have been associated with impaired mobility [18,19,20]. This suggests that these global measures of brain atrophy, whether related to aging, MS, or a combination of both, may significantly contribute to reduced mobility in older adults with or without MS.

The current cross-sectional study examined if central factors, measured by cognitive processing speed (Symbol Digit Modalities Test) and markers of global brain atrophy (gray matter volume, white matter volume, ventricular volume), were associated with mobility (Short Physical Performance Battery) in older adults with and without MS. We expected that all measures of central function would be associated with lower-extremity mobility; however, we expected these associations to be stronger in older adults with MS based on the greater extent of functional decline and atrophy in this group compared with older adults who do not have MS.

## 2. Methods

### 2.1. Participants

**Figure 1:**
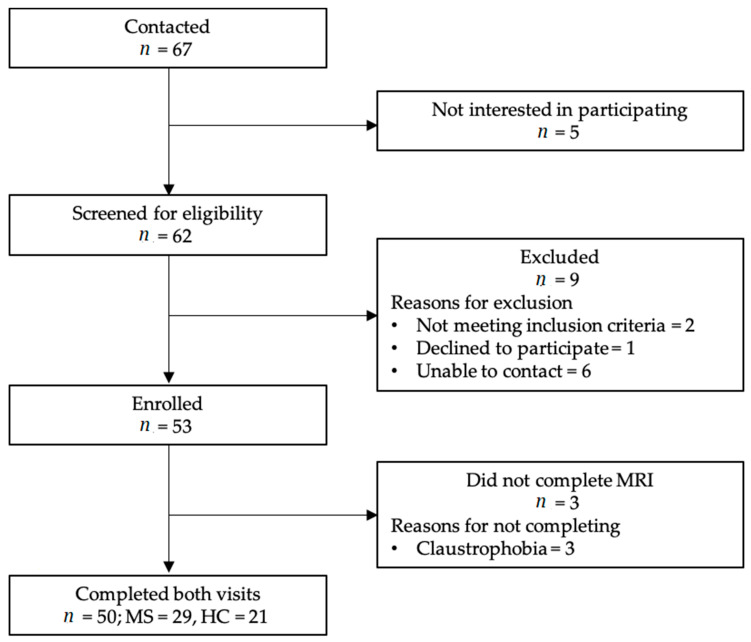
The flow of participants through the recruitment and enrollment process. Three individuals (2 MS, 1 HC) were unable to complete the MRI and, therefore, were excluded from any MRI-related analyses. MS = multiple sclerosis; HC = healthy control.

Participants were recruited between July 2018 and October 2020 using flyers distributed in the local community, previous research volunteers from studies in our laboratory, and word of mouth. Sample size for the current study was based on similar cross-sectional studies examining associations among cognitive function, brain structure, and mobility in adults with MS [21,22,23]. The inclusion criteria for participants with MS were diagnosis of MS, self-reporting as free of relapses over the previous 30 days, 55 years of age or older, able to walk independently or with an assistive device (individuals requiring the use of a wheelchair were excluded), and no contraindications to MRI (e.g., implanted metal devices). Control participants were age- and sex-matched, and inclusion criteria were the same for control participants except for the diagnosis of MS and being free of relapses in the previous 30 days. Additionally, control participants were required to be free of any neurologic disorder (e.g., Parkinson’s disease, stroke). Figure 1 provides a flow chart of the recruitment and enrollment process.

### 2.2. Measures

#### Physical Function

The Short Physical Performance Battery (SPPB) measures mobility based on a composite of standing balance, gait speed, and lower-extremity strength [24,25]. Standing balance was assessed by participants maintaining balance while standing with feet side-by-side, semi-tandem, and tandem for up to 10 s in each position. The tests were performed in a progressive manner based on successful completion of the previous position (i.e., 10 s of balance was achieved). Gait speed was assessed as the time to walk a 4 m distance at a normal, comfortable pace across two trials; overall gait speed was the average time of the trials. The chair stand test assessed lower-extremity strength. Participants were instructed to stand fully from a seated position and then return to sitting five times as quickly and safely as possible over one trial. Per SPPB protocol [24,26], each performance measure was scored from 0 (inability to complete the test) to 4 (highest level of performance), and the scores were summed to provide a final measure of lower-extremity function between 0 and 12; higher SPPB scores indicate better lower-extremity function. The SPPB is a valid measure of lower-extremity mobility in older adults with MS [3], and performance on the SPPB predicts disability in community-dwelling older adults [24,27]. The rates of developing disability within 1 year for those scoring 4 to 6 and 7 to 9 on the SPPB were 47% and 27%, respectively, among community-dwelling older adults without MS [27].

### 2.3. Cognitive Function

Cognitive impairment in MS often presents as slowed cognitive processing speed [28]; this cognitive domain was selected as a representative measure of cognitive function. Cognitive processing speed was measured using the Symbol Digit Modalities Test (SDMT) [29]. Participants were asked to match single number digits to a series of symbols based on a key provided at the top of the page. Responses were stated aloud by the participant and manually recorded by the test administrator. The final score was the number of correct pairings made within 90 s, and a higher score indicates better cognitive processing speed. The SDMT scores can be validly and reliably interpreted as a measure of cognitive processing speed in MS [30]. 

### 2.4. MRI Acquisition and Analysis

MRI acquisition was completed on a Siemens Prisma 3 Tesla whole-body MRI scanner (Malvern, PA, USA). We acquired high-resolution 3D T1-weighted structural brain images using a magnetization prepared, rapid acquisition gradient echo (MPRAGE) sequence and the following parameters: repretition time (TR) = 2100 ms, echo time (TE) = 3.41 ms, flip angle = 9°, effective inversion time (TI) = 900 ms, 256 × 256 matrix, field of view (FOV) = 256 mm, number of excitations (NEX) = 1176 slices, 1.00 mm slice thickness, and 0 mm skip. We removed all nonbrain tissue from the images using the Brain Extraction Tool [31] from FMRIB’s Software Library (FSL; Oxford, UK). Bias-field correction ensured accurate brain extraction. We then used FSL’s SIENAX [32,33] to compute normalized whole-brain gray matter volume (GMV), white matter volume (WMV), and ventricular CSF volume (vCSF) [34,35]. This approach for MRI analysis is consistent with previous studies that examined correlations with brain volumetry in young and middle-aged adults [21,22,23]; however, it should be noted that other approaches of image processing may yield slightly different results [36]. 

### 2.5. Procedures

The study protocol was approved through full-board review by a University Institutional Review Board. Written informed consent was provided by all participants. All study procedures were performed by highly trained laboratory personnel who were un-blinded regarding MS status; this was necessary for avoiding risks of injury during the administration of the SPPB. All participants completed a demographics questionnaire that included clinical characteristics for those with MS, the SPPB, and the SDMT during a single visit to the laboratory. During this visit, participants with MS further underwent a neurological evaluation by a Neurostatus-certified examiner for establishing disability status based on the Expanded Disability Status Scale (EDSS) [37]. The EDSS is an approach to measuring neurologic impairment in MS. Individual functional systems, namely brain stem, cerebellar, sensory, pyramidal, visual, cerebral, bowel and bladder, and ambulation, are assessed and scored, and an overall disability score ranging from 0 (normal) to 10 (death due to MS) is determined based on the functional system scores [37]. On a separate day after the initial visit, all participants underwent the MRI at a local university hospital. The laboratory visit was always completed prior to the MRI.

### 2.6. Data Analysis

The data were analyzed using SPSS Statistics version 25.0 (Armonk, NY, USA) with an unadjusted significance level of *p* < 0.05. Descriptive statistics for both groups are presented as mean (SD) unless otherwise noted. Group differences (i.e., older adults with MS vs. controls) for physical function (SPPB), cognitive function (SDMT), and MRI metrics (GMV, WMV, and vCSF volumes) were examined using independent samples *t*-tests. Standard z-scores were computed for the SDMT controlling for age, sex, and years of education [38]. Effect sizes (Cohen’s *d*) were generated for all variables to further examine the differences between the groups. Values of 0.2, 0.5, and 0.8 were interpreted as small, moderate, and large, respectively [39]. Spearman nonparametric correlations were used to examine the associations between physical function and central nervous system (CNS) measures (i.e., cognitive function and MRI metrics) [40]. We chose nonparametric correlations as these minimize the influence of non-normality and outliers. This has been recommended when analyzing MRI outcomes [41]. The magnitude of correlation coefficients was interpreted as small, medium, and large using values of 0.1, 0.3, and 0.5, respectively [39].

## 3. Results

The demographic and clinical characteristics of participants are provided in Table 1. The MS group (*n* = 31) and the healthy control group (*n* = 22) were similar in age, sex, race, and years of education. The older adults with MS predominantly had relapsing–remitting MS, a mean disease duration of 18.3 (6.1) years, and moderate disability based on a median EDSS score of 4.0 (IQR = 1.5).

Descriptive data and statistical analysis of mobility, cognitive function, and MRI metrics for older adults with MS and age- and sex-matched healthy controls (HC) are provided in Table 2. We were unable to obtain MRI data for two participants in the MS group and one HC participant. Mobility was significantly worse for older adults with MS than HC group (t(51) = −3.02, *p* = 0.004, *d* = 0.91). Cognitive function did not differ by group (t(51) = −0.72, *p* = 0.47, *d* = 0.20). The mean z-scores were −0.48 (1.03) for the MS group and −0.29 (0.99) for the healthy control group. Normalized GMV (NGMV) did not differ by group (t(48) = −0.28, *p* = 0.78, *d* = 0.08), whereas normalized WMV (NWMV) was lower (t(48) = −2.05, *p* = 0.047, *d* = 0.59) and normalized vCSF volume was higher (t(48) = 2.97, *p* = 0.005, *d* = −0.88) for the MS group than the HC group.

Correlations between physical function and CNS measures are provided for both groups in Table 3. There were no statistically significant correlations between physical function, assessed using the SPPB, and any CNS measures (cognitive function or whole-brain MRI metrics) for either group.

## 4. Discussion

The current study examined the influence of central factors on mobility in older adults with MS and a sample of age- and sex-matched healthy older adults. The primary results were that mobility, assessed by a composite measure that included balance, gait speed, and leg strength, was significantly worse in older adults with MS than the sample of older adults without MS and, in both groups, neither cognitive processing speed nor global measures of brain atrophy were associated with mobility, despite differences in white matter and ventricular volumes between the groups. This suggests that factors other than cognitive function and global brain atrophy may explain declines in mobility as people with or without MS age.

Mobility was significantly worse in older adults with MS than older adults without MS. This result confirms previous research demonstrating greater motor dysfunction in older adults with MS compared to older adults without MS [3,42]. It is further interesting that, while not a primary outcome in the current study, cognitive processing speed was not significantly different between groups in our samples of older adults with MS and age- and sex-matched controls. This result was not completely unexpected; previous research has indicated that the aging process may have a differential and complex effect on physical and cognitive functions in persons with MS [42,43]. This underscores the importance of research investigating this discrepancy between the effects of aging on physical and cognitive function in adults with MS.

Our results suggest that neither cognitive processing speed nor global measures of brain atrophy influenced mobility in older adults with or without MS. This is in contrast to our hypothesis that was based on evidence in comparatively younger adults with MS that demonstrated associations between measures of mobility and cognitive function [10,11,12] and brain atrophy [18,19,20]. Nevertheless, this result does not fully eliminate the possibility of central factors influencing mobility in aging adults with or without MS. Atrophy within focal brain regions that are more functionally relevant for mobility, such as the thalamus and basal ganglia, may have a significant effect on lower-extremity physical function in older adults with or without MS [44,45]. Additionally, there is a possibility that functional measures of CNS integrity may influence mobility in older adults with or without MS. For example, one recent study in adults with MS demonstrated an association between walking training and increased sensorimotor functional connectivity between the supplementary motor areas and the primary somatosensory cortices, suggesting the functional connectivity of this locomotor network contributes to mobility and should be considered in future research [46]. 

It further is possible that factors outside of the CNS have a greater influence on mobility in aging adults with or without MS. Indeed, it has been well documented that exercise training and lifestyle physical activity are associated with mobility in persons with MS across the lifespan [47,48]. This is supported by evidence in younger adults with MS that has demonstrated an association between aerobic capacity and walking performance [49]. The focal comparison of the influence of peripheral vs. central factors that may influence impaired mobility in aging individuals with or without MS is certainly warranted. Identifying the factors that have the greatest influence on mobility as people age would provide a target for interventions for improving mobility outcomes in this population.

Maintaining or improving mobility as adults with or without MS age is a personally and clinically significant outcome. Our results indicate that mobility is not associated with cognitive processing speed or global brain atrophy and suggest that other factors may influence mobility more in aging adults with or without MS. Other CNS measures such as functionally relevant subcortical gray matter structures and the functional connectivity of these structures should be considered. Additionally, the influence of peripheral factors such as cardiovascular fitness and lifestyle physical activity should be examined. Identifying the influential factors of mobility disability with aging in adults with and without MS is a necessary first step in developing effective rehabilitation approaches for improving mobility.

The current study has several notable limitations. First, the cross-sectional research design of the study allows for only correlational analyses and precludes the determination of causation. The current sample of adults with MS and age- and sex-matched controls were relatively young with moderate disability, and therefore, the results may not be generalizable to adults older than 65 years with more severe disability. We did not collect data on variables, such as depression and fatigue, that may influence cognitive function. Additionally, while the SPPB was chosen as the measure of mobility in the current study based on its validity as a measure of mobility in both older adults [24,27] and older adults with MS [3], it should be noted that it a composite score consisting of measures of balance, gait speed, and leg strength; therefore, we were unable to assess the associations of each of these components individually. Another important limitation to note is the uneven sample sizes between the MS and HC groups. This may introduce bias into the analyses.

## 5. Conclusions

Overall, the results of the current study suggest that neither cognitive function nor whole-brain measures of atrophy are significant contributors to mobility disability in older adults with and without MS. This does not support our hypothesis that was based on evidence from comparatively younger adults with MS, suggesting that the factors that influence mobility disability in aging adults with MS may be different than the factors that influence mobility disability in younger adults with MS.

## Figures and Tables

**Table 1:** Demographic and clinical characteristics.

Variable	MS (*n* = 31)	HC (*n* = 22)	*p*-Value
Age; years; mean (SD)	63.0 (5.8)	63.7 (5.7)	0.65
Sex			0.77
Women; *n* (%)	25 (80.6)	17 (77.3)	
Men; *n* (%)	6 (19.4)	5 (22.7)	
Race			0.18
Caucasian; *n* (%)	26 (83.9)	15 (68.2)	
African American; *n* (%)	5 (16.1)	7 (31.8)	
Education; years; mean (SD)	16.3 (2.2)	16.8 (2.8)	0.45
MS type			
RRMS; *n* (%)	29 (93.5)		
Progressive; *n* (%)	2 (6.5)		
Disease duration; years; mean (SD)	18.3 (6.1)		
EDSS; median (interquartile range)	4.0 (1.5)		
DMT use			
Yes; *n* (%)	24 (77.4)		
No; *n* (%)	7 (22.6)		

MS = multiple sclerosis, HC = healthy control, RRMS = relapsing–remitting MS, EDSS = Expanded Disability Status Scale, DMT = disease-modifying therapy.

**Table 2:** Mean values for mobility, cognitive processing speed, and whole-brain MRI metrics.

Variable	MS	HC	*p*-Value	Effect Size (*d*)
Mobility (SPPB)	10.3 (2.4)	11.8 (0.5)	0.004 *	0.91
Cognitive processing speed (SDMT)	48.0 (9.6)	49.9 (9.2)	0.47	0.20
NGMV (mm^3^)	739,043 (45,692)	742,860 (48,998)	0.78	0.08
NWMV (mm^3^)	702,842 (51,955)	731,757 (45,564)	0.047 *	0.59
vCSF (mm^3^)	60,101 (25,721)	41,095 (16,426)	0.005 *	−0.88

* = *p* < 0.05 indicating a significant difference between the groups. Effect sizes between the groups indicate the magnitude of the effect of MS on the measure. Higher scores on the SPPB and SDMT indicate greater mobility and cognitive processing speed, respectively. MS = multiple sclerosis, HC = healthy control, SPPB = Short Physical Performance Battery, SDMT = Symbol Digit Modalities Test, NGMV = normalized gray matter volume, NWMV = normalized white matter volume, vCSF = ventricular cerebrospinal fluid volume.

**Table 3:** Correlations between central factors and mobility.

	Mobility (SPPB)	
Variable	MS	HC
Cognitive processing speed (SDMT)	0.26	0.08
NGMV (mm^3^)	−0.30	−0.40
NWMV (mm^3^)	−0.11	−0.24
vCSF (mm^3^)	−0.06	0.16

All correlations are Spearman nonparametric correlations. For all correlations, *p* > 0.05, suggesting that neither cognitive function nor global brain structure is associated with mobility in older adults with or without MS. MS = multiple sclerosis, HC = healthy control, SPPB = Short Physical Performance Battery, SDMT = Symbol Digit Modalities Test, NGMV = normalized gray matter volume, NWMV = normalized white matter volume, vCSF = ventricular cerebrospinal fluid volume.

## Data Availability

The data presented in this study are available on request from the corresponding author. The data are not publicly available due to institutional guidelines.
